# Phenotypic Variability of Osteogenesis Imperfecta Type V Caused by an *IFITM5* Mutation

**DOI:** 10.1002/jbmr.1891

**Published:** 2013-06-18

**Authors:** Jay R Shapiro, Caressa Lietman, Monica Grover, James T Lu, Sandesh CS Nagamani, Brian C Dawson, Dustin M Baldridge, Matthew N Bainbridge, Dan H Cohn, Maria Blazo, Timothy T Roberts, Feng-Shu Brennen, Yimei Wu, Richard A Gibbs, Pamela Melvin, Philippe M Campeau, Brendan H Lee

**Affiliations:** 1 Department of Bone and Osteogenesis Imperfecta, Kennedy Krieger Institution, Johns Hopkins UniversityBaltimore, MD, USA; 2 Department of Molecular and Human Genetics, Baylor College of MedicineHouston, TX, USA; 3 Human Genome Sequencing Center, Baylor College of MedicineHouston, TX, USA; 4 Department of Structural and Computational Biology & Molecular Biophysics, Baylor College of MedicineHouston, TX, USA; 5 Department of Molecular, Cell and Developmental Biology, University of California-Los AngelesLos Angeles, CA, USA; 6 Department of Orthopaedic Surgery, University of California-Los AngelesLos Angeles, CA, USA; 7 Division of Medical Genetics, Scott & White Memorial Hospital, Texas A&M Health Science Center, College of MedicineTemple, TX, USA; 8 Howard Hughes Medical InstituteHouston, TX, USA

**Keywords:** OSTEOGENESIS IMPERFECTA, IFITM5, UNTRANSLATED REGION, HYPERPLASTIC CALLUS

## Abstract

In a large cohort of osteogenesis imperfecta type V (OI type V) patients (17 individuals from 12 families), we identified the same mutation in the 5′ untranslated region (5′UTR) of the interferon-induced transmembrane protein 5 (*IFITM5*) gene by whole exome and Sanger sequencing (*IFITM5* c.–14C > T) and provide a detailed description of their phenotype. This mutation leads to the creation of a novel start codon adding five residues to IFITM5 and was recently reported in several other OI type V families. The variability of the phenotype was quite large even within families. Whereas some patients presented with the typical calcification of the forearm interosseous membrane, radial head dislocation and hyperplastic callus (HPC) formation following fractures, others had only some of the typical OI type V findings. Thirteen had calcification of interosseous membranes, 14 had radial head dislocations, 10 had HPC, 9 had long bone bowing, 11 could ambulate without assistance, and 1 had mild unilateral mixed hearing loss. The bone mineral density varied greatly, even within families. Our study thus highlights the phenotypic variability of OI type V caused by the *IFITM5* mutation.

## Introduction

Osteogenesis imperfecta (OI) is a genetically and clinically heterogeneous disorder of connective tissues.^1^ Although originally classified by phenotype and inheritance as four distinct types by Sillence and colleagues^2^ in 1979, recent advances in mutation analysis and the recognition of new phenotypes has resulted in an extended classification.^2^,^3^ First reported by Glorieux and colleagues^4^ in 2000, OI type V is a nonlethal autosomal dominant form of OI. There are an estimated 25,000 cases of OI in the United States, 4% to 5% of which may be type V.^3^,^5^ As with other nonlethal forms of the disorder, such as Sillence types I, III, and IV, OI type V has been characterized by moderate bone fragility, scoliosis, long-bone deformities, and diminished stature. However, type V demonstrates highly variable expressivity. It is distinguished from other OI types by the frequent occurrence of HPC after fracture or surgery in approximately 65% of the affected individuals, the presence of bilateral radial head dislocation, and ossification of the interosseous membrane in the forearm and lower extremity. However, type V patients typically do not have discoloration of sclerae or dentinogenesis imperfecta (DI).^4^

At the molecular level, OI type V patients lack detectable mutations in the *COL1A1* and *COL1A2* genes that are found in more than 90% of cases of OI types I through IV or in recessive OI genes.^6^ In 2012, it was reported that a mutation in the 5′ untranslated region (5′UTR) of the *IFITM5* gene (*IFITM5* c.–14C > T) can cause OI type V.^7^,^8^ Interferon-inducible transmembrane protein 5 (IFITM5), otherwise known as bone restricted ifitm-like protein (BRIL), is a two-transmembrane domain membrane protein that presents its termini extracellularly. Its expression is limited to osteoblasts and shows a pattern similar to the transcription factor osterix (SP7).^9^–^11^ It has been shown that IFITM5 plays a role in mineralization in vitro and is involved in bone growth during prenatal development in a mouse model.^10^,^11^

Our report confirms the presence of the same mutation (*IFITM5* c.–14C > T) in our cohort and describes the clinical and radiological manifestations of the disease in 17 patients, approximately one-half of whom have inherited the disorder from an affected parent.

## Patients and Methods

### Patients

Permission to report this retrospective chart analysis was granted by the Institutional Review Board at the Kennedy Krieger Institute/Johns Hopkins University. Molecular testing was performed according to the specifications of the Baylor College of Medicine Institutional Review Board.

Thirteen OI type V patients were identified in a review of approximately 350 OI patients at the Kennedy Krieger Institute; thus, OI type V patients constitute 3.7% of the center’s OI population. Four other patients were enrolled at the Skeletal Dysplasia Clinic of the Texas Children’s Hospital. Ten patients had Sanger sequencing at Clinical Laboratory Improvement Amendments (CLIA)-certified laboratories by Sanger sequencing for *COL1A1* and *COL1A2* and were not found to harbor mutations in these genes. Three patients did not have any molecular studies prior to enrollment (patients 6, 7, and 13); they were diagnosed by their archetypical clinical and radiographic presentation.

Radiographic images and reports were reviewed for the presence of radial head dislocation and mineralized interosseous membranes between the radius and ulna, and/or the tibia and fibula. Evidence was sought for excessive callus formation postinjury, which is defined as a relatively large, radio-opaque lesion originating from the surface of the bone, showing unusual features such as sun-ray spicules or a “butterfly-like” appearance.^12^ Also, spine X-rays were reviewed for presence of scoliosis. Comparative *Z*-scores for height and weight were obtained from the Center for Disease Control and Prevention’s 2004 Advanced Data Vital and Health Statistics.^13^ Dual-energy X-ray absorptiometry (DXA) bone density measurements of lumbar vertebrae were obtained using a Hologic DelphiA machine (software version 11.2:3; Hologic, Bedford, MA, USA).

### Exome sequencing

Before the publication of the gene for OI type V, exome sequencing and analysis was conducted as previously described to try to identify the causative gene.^14^ Briefly, exomes were captured on NimbleGen’s SeqCap EZ V2.0 library (Roche NimbleGen, Inc., Madison, WI, USA) and sequencing was conducted on Illumina HiSeq2000 (Illumina, San Diego, CA, USA). Reads were aligned with Burrows-Wheeler Aligner (BWA) to human reference genome GRCh37/hg19 and locally realigned with the Genome Analysis Toolkit (GATK) around potential insertions or deletions (INDELs). Single-nucleotide polymorphisms (SNPs) were called using Samtools Pileup (version 0.1.17)^15^ and short indels were called using Samtools,^15^ Atlas-INDEL,^16^ and GATK.^17^ Variants were annotated with ANNOVAR (http://www.openbioinformatics.org/annovar)^18^; protein-impacting variants that were rare (minor allele frequency <5%) or novel were preferentially explored.

### Sanger sequencing

Amplicons were generated using 1 ng/µL of genomic DNA using TaqMan polymerase (ABI, Life Technologies, Carlsbad, CA, USA) following the manufacturer’s protocol with an annealing temperature of 60°C and an amplification of 1 minute. Primers used were forward: 5′-AGGGCGACAGGGCTATAAGTGAG-3′ and reverse: 5′-GAAGCCGAGGCAACACAGATTCAGGTAG-3′. Products were verified by agarose gel then sequenced by Sanger sequencing using the same primers at Beckman Coulter Genomics (Danvers, MA, USA).

## Results

Demographic data and treatment modalities are summarized in Table 1. Four families with multiple individuals with OI type V are grouped in Table 1; the remaining 8 nonfamilial cases were the result of spontaneous mutations. The average age at diagnosis in our cohort of 8 males and 9 females was 1 year of age. *Z*-scores for height averaged −2.9 ± 3.4 for males and −4.5 ± 3.6 for females (mean values are presented with standard deviations throughout this work). *Z*-scores for weight averaged −1.5 ± 1.4 for males and −1.3 ± 1.0 for females. Body mass index (BMI) values averaged 29 ± 8 kg/m^2^ for men and 29 ± 2 kg/m^2^ for women over the age of 18 years.

**Table 1:** Demographic Overview of Cohort

Patient	Gender	Age at OI Dx (years)	Age at examination (years)	Ambulatory status	Height (cm)	Height *Z*-score	Weight (kg)	Weight *Z*-score	BMI (kg/m^2^)	Race^a^	Familial association	Bisphosphonate treatment
1	M	Birth	7	Ambulatory	118	−0.4	19	−1.1	13	A/C	Son of patient 3	Pamidronate (IV)
2	M	1.5	12	Ambulatory	149	0.2	36	−0.4	16	A/C	Son of patient 3	Pamidronate (IV)
3	F	0.5	48	Ambulatory w/assistance	147	−2.9	61	−0.4	28	C	Mother of patients 1 and 2	Alendronate (PO)
4	F	2.0	43	Wheelchair	97	−11.9	24	−1.6	26	C	–	–
5	F	2.0	33	Ambulatory	115	−6	41	−0.9	31	C	–	–
6	F	In utero (27 weeks)	2 months	n/a	53	−2.4	5	−0.8	17	C	Daughter of patient 7	–
7	M	Birth	32	Ambulatory	147	−3.6	43	−1.2	20	C	Father of patient 6	–
8	M	0.5	60	Wheelchair	98	−10.4	33	−3.6	34	C	Father of patient 9	Pamidronate (IV) and alendronate (PO)
9	M	0.7	24	Ambulatory	173	−0.5	68	−0.4	23	A/C	Son of patient 8	Pamidronate (IV)
10	F	2.0	23	Ambulatory	151	−1.5	74	−0.4	32	C	–	–
11	M	3.0	23	Wheelchair	152	−3.4	91	0.5	39	C	–	–
12	F	1.3	5	Ambulatory	86	−4.3	10	−1.8	13	C	–	Pamidronate (IV)
13	M	0.7	47	Wheelchair	150	−2.5	54	−2.2	24	C	–	–
14	M	n/a	6	Ambulatory	101	−3.0	14	−3.2	14	C	–	Alendronate
15	F	Birth	36	Ambulatory	140		56		28	C	–	Alendronate; ibandronate; zoledronic acid; calcitonin; teriparatide
16	F	1	4	Ambulatory	91	−2.7	12	−3.1	14	C	Daughter of patient 17	Pamidronate (IV)
17	F	0.5	21	Ambulatory	147		64		29	C	Mother of patient 16	None

OI = osteogenesis imperfecta; Dx = diagnosis; BMI = body mass index; IV = intravenously; PO = orally; n/a = not available.

a A = Asian; C = Caucasian; H = Hispanic.

Clinical features are summarized in Table 2. Two patients exhibited blue sclerae. Three patients had pes planus. Eleven patients were described as having bilateral hyperextensible joints in at least one extremity, typically in digits and elbows. Fifteen patients exhibited limited/absent ability to supinate and pronate the forearm due to dislocation of the radial head. With regard to other common OI findings, triangular facies were observed in 10 patients. DI was not observed in any patient. Hearing loss, either conductive or mixed, was generally absent from the cohort; only 1 patient (patient 2) was noted to have mild (8 kHz) unilateral mixed hearing loss.

**Table 2:** Clinical Features of Cohort

Patient	Blue sclera	Triangular facies	Pes planus	Hyperextensible joints	Limited range supination or pronation	COL1A1/COL1A2 analysis for mutation	*IFITM5* c.–14C > T mutation
1	−	+	+	−	Limited	Negative	+
2	−	+	+	+ (knees, elbows)	Limited	Negative	+
3	−	+	−	+ (digits)	Absent	Negative	+
4	−	+	−	+ (digits)	Absent	Negative	+
5	−	+	−	+	Limited	Negative	+
6	−	−	−	−	Normal	Not performed	+
7	−	+	−	−	Limited	Not performed	+
8	−	+	−	−	Absent	Negative	+
9	−	−	−	+ (digits)	Limited	Negative	+
10	−	+	+	−	Limited	Negative	+
11	−	−	−	+ (elbows)	Limited	Negative	+
12	−	+	−	+ (ankles, hips)	Limited	Negative	+
13	−	−	−	+ (elbows)	Absent	Not performed	+
14	−	−	−	+ (elbows)	Normal	Negative	+
15	−	+	−	+	Absent	Negative	+
16	+	−	−	+	Limited	Negative	+
17	+	−	−	−	Limited	Not performed	+

**Figure 1:**
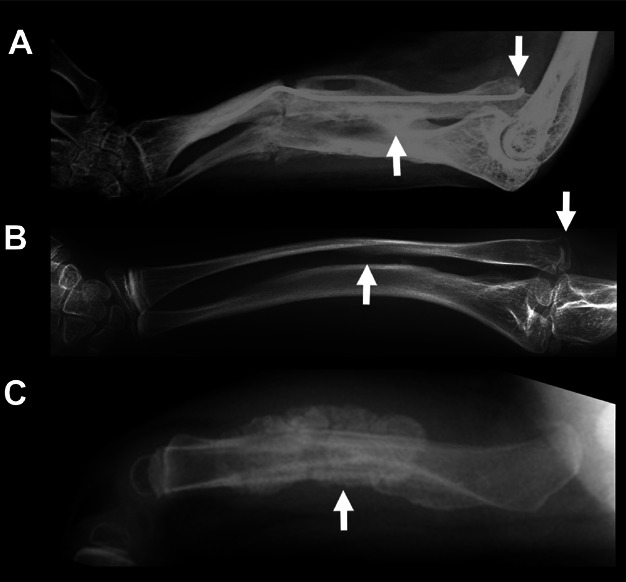
Radiographic features in OI type V. (*A*, *B*) Radial head dislocation (arrows on the right) and forearm interosseous membrane calcification (middle arrows) in patients 1 and 3, respectively. (*C*) HPC in patient 14 at 1.5 years.

Radiographic findings characteristic of OI Type V patients are summarized in Table 3. Thirteen patients exhibited interosseous membrane calcification (Fig. 1). One patient (patient 5) was incidentally noted to have calcification of her left lower extremity interosseous membrane. Also, 14 patients were observed to have dislocated radial heads bilaterally. Thirteen patients had scoliosis; all patients over the age of 5 years exhibited mild to severe scoliosis. Additionally, 9 patients exhibited bowing of long bones: 6 had anterior bowing of the tibias and/or fibulas, 3 had bowing of the femurs, and 6 had bowing of the forearms. Ten patients experienced one or more episodes of HPC formation (Fig. 1) after trauma-related fractures.

**Table 3:** Radiographic Findings of Cohort

Patient	Calcification of interosseous membranes	Radial head dislocation	HPC	Scoliosis
1	+	+	+ (humerus)	+
2	n/a	+	−	+
3	+ (fusion)	+	−	+
4	+	+	+ (femur)	+
5	+	+	−	+
6	−	−	+ (ribs)	−
7	+ (fusion)	+	+ (femur)	+
8	+	+	−	+
9	+	+	−	+
10	+	+	+ (femur)	+
11	n/a	+	−	+
12	+	+	+	−
13	+	+	+ (humerus)	+
14	n/a	−	+	−
15	+	No X-ray but supination/pronation absent	−	+
16	+	+	+ (femur)	+
17	+	+	+ (femur)	−

HPC = hyperplastic callus; n/a = not available.

**Figure 2:**
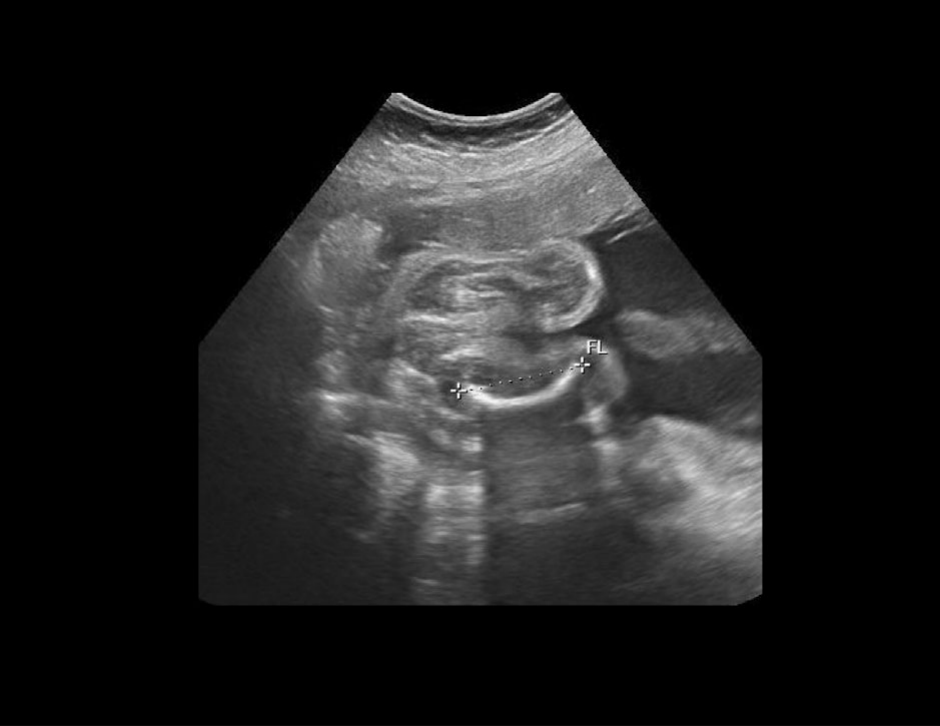
Prenatal ultrasound at 22 weeks showing bowing of the femur (the crosshairs show the extremities of the femur).

An unusual opportunity permitted us to follow the course of pregnancy in the first child of a 35-year-old man with OI type V. The ultrasound scans at 22 weeks of gestation permitted the detection of skeletal abnormalities (bowing of the femur illustrated in Fig. 2), and at 33 weeks a thin calvarium and angulated ribs suggesting intrauterine fracture. At birth, the infant had two healing rib fractures, bowing of the femurs, and a thin calvarium but no other abnormalities.

Extraskeletal findings of note include 2 patients with mitral valve prolapse (patients 8 and 10), 2 patients (patients 7 and 8) with histories of symptomatic nephrolithiasis, and 1 patient with hypertension and depression (patient 15). Orthopedic surgical histories include 7 patients who had undergone at least one long-bone intramedullary rodding (typically tibias and femurs). Additionally, 3 patients underwent posterior spinal fusion for scoliosis. Surgical resection of dislocated radial heads were performed in 2 patients from the same family (patients 2 and 3) with only marginal improvements noted in elbow range of motion and no increase in range of motion at the wrist. There was no HPC formation following rodding or other orthopedic surgeries in our cohort.

Of the 17 patients with OI reported here, 10 received treatment with bisphosphonates: 5 received only pamidronate intravenously (patients 1, 2, 9, 12, and 16), 1 adult (patient 3) and 1 pediatric patient (patient 14) received only oral alendronate, and another 2 adults (patients 8 and 15) received both oral and intravenous treatments in succession (alendronate and pamidronate or zoledronic acid).

The results of clinical laboratory studies of serum calcium, phosphorus, 25(OH) vitamin D, and insulin-like growth factor were within normal limits for all patients. Alkaline phosphatase activity, although elevated during fracture or HPC formation, was typically within normal limits with one notable exception: serum total and bone-specific alkaline phosphatase activities were persistently elevated in 3 patients.

Exome sequencing was performed for 17 individuals before the reports on the gene identification were published. Over 90% of the target region obtained over 20× coverage, with an average coverage between 80× and 130× for coding regions. Exome sequencing identified on average 574 rare variants per individual predicted to impact proteins (missense, nonsense, indels, and splice-site mutations). No mutations were identified in the OI genes *COL1A1*, *COL1A2*, *CRTAP*, *LEPRE1*, *PPIB*, *FKBP10*, *SERPINH1*, *SERPINF1*, *PLOD2*, or *SP7*. When considering only coding regions and variants within five positions of splice sites, exome sequencing identifies on average 300 to 500 rare or novel coding variants in any individual.^19^ In our cohort, no gene showed rare or novel variants in more than three families, suggesting either great genetic heterogeneity or that the causative mutation was not captured by the sequencing or was missed by our analysis, which did not include untranslated regions. After the publication of a de novo mutation introducing an early start site in *IFITM5*,^7^,^8^ this region was analyzed on the exome data. The average coverage was 19× within 5′UTRs and 12× within 3′UTRs. Exome sequencing data showed the same mutation in our cohort of OI type V individuals. We confirmed the mutation by Sanger sequencing and the same mutation was present in 12 families (17 individuals). This experience highlights the importance of considering UTRs in analyzing exome data.

## Discussion

In 2000, Glorieux and colleagues^4^ described OI type V as a phenotype different from Sillence types I to IV. Definition for this subtype was based on the recognition of the frequent occurrence of HPC in a group of patients that were initially categorized as Sillence type IV disease. Subsequent literature includes a cohort study on 12 Korean patients,^20^ a summary of the response to treatment with bisphosphonates,^21^ a description of the natural history of HPC in type V patients,^22^ and just recently, the identification of the gene by two groups.^7^,^8^

Clinical and radiological findings in our cohort of 17 patients strengthen the case for considering this as a separate phenotype within the OI family. Although hearing loss—a symptom reportedly experienced by approximately 50% of type I OI adults—appears to occur infrequently in OI type V, its real frequency cannot be estimated in the absence of audiological testing or without a larger number of patients.^23^,^24^ Only 1 patient in our cohort experienced minimal unilateral sensorineural hearing loss.

**Figure 3:**
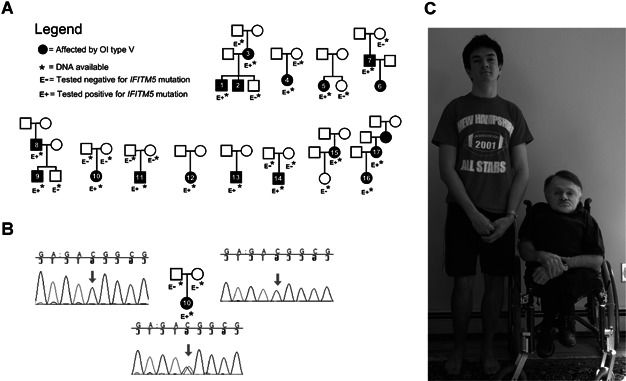
(*A*) Pedigrees of the individuals described. (*B*) Example of Sanger sequencing results for the *IFITM5* mutation, here in patient 10 and her parents. (*C*) Two affected members of the same family: patients 8 and 9. The father is aged 52 years, and the son is 18 years old at the time of the photograph. Although the son was treated at an earlier age with bisphosphonates, this does not explain the important difference in phenotype.

Variable expressivity, both within and between affected families, is characteristic of OI type V. Illustrative of the diverse phenotype, over one-half of these patients were able to ambulate unassisted whereas others required walking aids or wheelchairs. A photograph depicting the remarkably varied phenotypic expression of the same mutation in 2 affected members of the same family is seen in Fig. 3.

HPC is described as a postoperative or postfracture soft-tissue mass that may be confused with osteosarcoma because of an accompanying inflammatory component. CT and MRI findings characterize HPC as exuberant bone formation, disproportionate to the size of the affected bone with extensive expansion beyond the region of fracture.^12^ The differential diagnosis includes intraosseous osteosarcoma with aggressive periosteal reaction, periosteal osteosarcoma, juxtacortical myositis ossificans, and large osteochondromata.^12^ However, in the 10 type V patients who experienced one or more occurrence of HPC the hyperplastic response did not appear as a tumor mass. Rather, the hyperplastic response was usually confined to localized overgrowth of bone at the fracture site seen on X-ray rather than presenting as a soft tissue mass (Fig. 1). Our patients experienced callus formation in their femurs, humeri, and ribs. Similar to our findings, Cheung and colleagues^25^ reported a 65% incidence of HPC in OI type V patients, with one-half of the reactions occurring on or around the femurs. HPC has also been reported to involve the pelvis and forearms.^12^ In our cohort, HPC appeared to start from the periosteal region. In 1 patient (patient 5), postfracture calcification involved the plane between leg muscle bundles.

Another common radiographic finding in OI type V patients is the idiopathic calcification of the forearm interosseous membranes. The majority of our patients exhibited the mineralization phenomenon. Ossification was radiographically apparent in 1 patient by the age of 4 years. Images of the child’s arm can be seen in Fig. 1. Although it may be assumed that calcification at these sites follows trauma, the relationship, if any, of the mechanism underlying interosseous membranes mineralization and hyperplasic callus is unknown. In this series, the two processes were not linked, in that ossification of the interosseous membrane between the radius and ulna and tibia and fibula were not coincident with the existence of HPC in the same patients. A question arises as to why HPC occurs in only about 50% of type V patients and, further, why is this absent in other OI syndromes?

Congenital radial head dislocation (CRHD) can occur as an isolated abnormality or in at least 14 syndromes.^26^ Previously, it has been reported that the incidence of one or more radial head dislocations is 58% in type V patients, compared to an incidence of 1.5%, 8.5%, and 6.5% for types I, III, and IV, respectively.^27^ Dislocation of the radial head was observed in 0% to 29% of OI patients other than type V and in 86% of type V patients by Fassier and colleagues.^27^ Bilateral dislocation of the radial heads was observed in 88% of our patients. These patients have restricted pronation/supination of the forearm, a decreased flexion arc, and variable degrees of functional impairment. It is of interest that radial head dislocation was not present in the infant (patient 6) at age 4 months. Dislocation of the radial head is associated with significant functional impairment: Resection of the radial head has been proposed to improve function. However, only minimal improvement was observed in the flexion arc at the elbow, and no improvement occurred in the degrees of pronation or supination in the 2 patients who underwent radial head resection. Lack of improvement in forearm function following radial head excision was also reported by Lee and colleagues.^20^ Thus, it remains to be decided whether radial head excision should be recommended.

Glorieux and colleagues^4^ described the presence of appendicular metaphyseal bands as a “constant feature in growing type V patients.” These radio-dense bands are visualized on plain films located immediately adjacent to the growth plate, typically involving the proximal tibia, distal femur, or radius. No metaphyseal banding pattern was observed in this group. Last, it is worth noting that metaphyseal banding is described in OI type V patients should not be confused with the bisphosphonate-induced epiphyseal “zebra lines,” as are typically seen with cyclical IV pamidronate therapy.

Three OI type V patients were observed to have chronically elevated alkaline phosphatase values. In the case of 2 patients who were brothers, elevated alkaline phosphatase levels were seen even during bisphosphonate therapy, whereas their affected mother and normal father had normal alkaline phosphatase values. Serum alkaline phosphatase results were not reported by Lee and colleagues^20^ in 12 OI type V patients from three Korean families. Glorieux and colleagues^4^ observed that serum alkaline phosphatase increased markedly during periods of active HPC formation. However, Zeitlin and colleagues,^21^ when reporting the effect of intravenous pamidronate treatment in type V patients, observed initial alkaline phosphatase values to be above the upper level of normal (300 U/L) at 436 ± 160 U/L. Following 2 years of pamidronate treatment, alkaline phosphatase values declined approximately 50% from baseline, but less than the posttreatment decline in urinary N-telopeptide. Thus, the clinical significance of alkaline phosphatase elevation remains to be elucidated.

Numerous reports have suggested that pediatric-aged OI patients increase vertebral bone mineral density and decrease fracture rate in response to bisphosphonate treatment.^28^,^29^ In a recent report of 11 type V pediatric patients undergoing 2 years of cyclic pamidronate treatment, Zeitlin and colleagues^21^ reported a statistically significant lower incidence of fracture, increases in transiliac cortical thickness, and increases in lumbar vertebral body size and volumetric bone mass density.

The last decade has seen remarkable progress in both our clinical and molecular understanding of OI.^30^ Case finding of OI type V has been hampered by the marked phenotypic variability and until recently by the absence of a defined molecular cause. In turn, these have limited our ability to understand the mechanisms underlying the distinctive features of this syndrome: HPC formation, calcification of the interosseous membrane, and dislocation of the head of the radius. However, it is evident that OI type V involves aberrant mineralization that is not seen in other OI types associated with abnormal synthesis of type I collagen. IFITM5 is involved in both mineralization and bone growth, but very little is known about its function.^9^–^11^ The mechanism by which the *IFITM5* mutation cause the OI type V phenotype is unknown. We speculate that it might be related to an acquired molecular function due to the elongation of the protein, such as sustained signaling through an important osteogenesis signaling cascade.

The identification of the same mutation in 34 previous OI type V families^7^,^8^,^31^ and in these 12 families contrasts markedly with the wide distribution of type I collagen mutations in other OI phenotypes. IFITM5 is a 14.8-kDa protein that has two transmembrane domains with both the amino and carboxy terminus present extracellularly. These termini exhibit the least conservation within the IFITM family of proteins, and are the regions that most likely interact with other proteins.^11^,^32^ The first functional studies of IFITM5, also termed Bril, included expression studies by Northern blotting and in situ hybridization, both suggesting a bone-specific expression pattern.^10^,^11^ In vitro studies suggest a role in mineralization, including expression during osteoblast differentiation as well as increased mineralization in an overexpression model.^10^,^11^ Knockout mice show a possible defect in prenatal bone development, because newborn mice display shorter bones that are sometimes bent in appearance. However, these skeletal phenotypes were not present in adult mice.^10^ Furthermore, IFITM5 has only one known binding partner, FKBP11. It has been shown that the binding of IFITM5 to FKBP11 disrupts binding of CD9 with the FKBP11-CD81-CD9/FPRP complex and increases expression of interferon-induced genes.^9^,^10^ Thus, there may be an immune component to OI type V clinical manifestations.

We suspect that IFITM5 not only has a role in bone development but also may play a role in the immune response in bone. This mutation extends the amino-terminal extracellular domain by five amino acids and it is likely that this mediates protein interactions with IFITM5. It is possible that not only binding of FKBP11 may be altered, but also the downstream interferon response. It is also likely that IFITM5 interacts with other proteins in vivo and the mutation might increase, decrease or change this binding pattern. Through this process, it is likely that other components of osteogenesis are affected by the mutation and would lead to the low bone mass phenotype. Conditions under fracture repair may be different owing to the immune response and thus have an opposite phenotype, therefore increasing bone formation without proper feedback on fracture repair. Introducing the mutation in transgenic mice should allow the dissection of the mechanisms leading to osteogenesis imperfecta and hyperplastic callous formation.

## Disclosures

All authors state that they have no conflicts of interest.
